# Safety and Efficacy of Cisplatin and Doxorubicin Pressurized Intraperitoneal Aerosolized Chemotherapy (PIPAC) in Patients with Ovarian Cancer with Peritoneal Metastases: A Multicenter US Phase I Trial

**DOI:** 10.1245/s10434-025-18432-0

**Published:** 2025-09-30

**Authors:** Brad Nakamura, Rosemary Senguttuvan, Nora H. Ruel, Paul H. Frankel, Susan E. Yost, Sarah Cole, Sue Chang, Alexander Jung, Melissa Eng, Raechelle Tinsley, Timothy Synold, Daphne Stewart, Edward Wang, Joshua Cohen, Jeannine Villella, Richard L. Whelan, Amit Merchea, Danielle K. DePeralta, Yanghee Woo, Mustafa Raoof, Thanh Hue Dellinger

**Affiliations:** 1 Department of Surgery, City of Hope National Medical Center, Duarte, CA USA; 2 Department of Computation and Quantitative Medicine, City of Hope National Medical Center, Duarte, CA USA; 3 Department of Medical Oncology and Therapeutics Research, City of Hope National Medical Center, Duarte, CA USA; 4 Clinical Protocol Development, City of Hope National Medical Center, Duarte, CA USA; 5 Department of Pathology, City of Hope National Medical Center, Duarte, CA USA; 6 Department of Diagnostic Radiology, City of Hope National Medical Center, Duarte, CA USA; 7 Department of Surgery, Northwell Health, New York, NY USA; 8 Department of Surgery, Mayo Clinic Florida, Jacksonville, FL USA

**Keywords:** Pressurized intraperitoneal aerosolized chemotherapy, PIPAC, Ovarian cancer, Peritoneal metastases, Cisplatin, Doxorubicin

## Abstract

**Background:**

Pressurized intraperitoneal aerosolized chemotherapy (PIPAC) is a novel, minimally invasive method of delivering intraperitoneal chemotherapy with promising peritoneal disease control in ovarian cancer.

**Methods:**

This US multicenter prospective phase I trial (NCT04329494) evaluated the safety and efficacy of PIPAC cisplatin 10.5 mg/m^2^ and doxorubicin 2.1 mg/m^2^ (PIPAC-CD) every 6 weeks in ovarian cancer at three US centers. Primary endpoints were dose-limiting toxicities and adverse events. Secondary endpoints included response according to RECIST (Response Evaluation Criteria in Solid Tumors) criteria, laparoscopic peritoneal carcinomatosis index, histologic peritoneal regression grading score, progression-free survival (PFS), and overall survival (OS).

**Results:**

In total, 15 patients were enrolled. The median prior lines of therapy was 3 (range 1–10). The PIPAC completion rate (≥2 PIPACs) was 86.7%. A total of 76.9% of patients had extraperitoneal disease at baseline. One patient discontinued treatment for toxicity because of deterioration of her baseline Eastern Cooperative Oncology Group 2 performance status. There was one grade 3 abdominal pain, one grade 3 anorexia, and no grade 4 or 5 adverse events. Laparoscopic best response (peritoneal carcinomatosis index) and histologic response (peritoneal regression grading score) occurred in 30.8% and 46.2%, respectively. Radiologic best response (RECIST) was 6.7%, with one partial response and a stable disease rate of 26.7%. Median PFS and OS were 2.3 months (95% confidence interval 1.7–3.2) and 17.1 months (95% confidence interval 5.6–not reached), respectively (n=15).

**Conclusions:**

PIPAC-CD is feasible, safe, and well tolerated at academic US centers. OS and PFS were limited in patients with heavily pretreated ovarian carcinoma who underwent PIPAC-CD. Future trials should focus on optimizing PIPAC drug combinations and determining optimal patient selection criteria for ovarian cancer.

**Supplementary Information:**

The online version contains supplementary material available at 10.1245/s10434-025-18432-0.

The peritoneum is the most common site of metastasis and recurrence in ovarian carcinomas. Peritoneal metastases (PM) frequently cause ascites, malignant bowel obstruction, and urinary obstruction, resulting in reduced quality of life (QOL) and increased morbidity and mortality. PM are often unresectable and difficult to treat with traditional systemic therapy because of the limited drug uptake and drug distribution to the peritoneum.^1^ Regional therapies are efficacious in ovarian cancer (OC), including normothermic intraperitoneal chemotherapy and hyperthermic intraperitoneal chemotherapy (HIPEC) in optimally cytoreduced patients.^2–5^ However, in the recurrent setting, the role for intraperitoneal therapy is limited to optimal cytoreduction indications. Thus, novel regional therapies are needed to address recurrent, unresectable peritoneal metastases in OC.

PIPAC is an innovative chemotherapy delivery method to the intraperitoneal space that does not require optimal cytoreductive surgery. It aerosolizes chemotherapy through a nebulizer device delivered from a high-pressure injector at the time of diagnostic laparoscopy. This combination of aerosolization and increased intraabdominal pressure from pneumoperitoneum improves drug distribution throughout the peritoneal cavity and increases the depth of penetration in peritoneal tumors.^6^ In contrast to HIPEC, PIPAC can be frequently repeated and is well tolerated. The safety of PIPAC has been demonstrated in multiple international clinical trials.^7–12^ PIPAC cisplatin 10.5 mg/m^2^ and doxorubicin 2.1 mg/m^2^ (PIPAC-CD) was endorsed by the International Society for the Study of Pleura and Peritoneum as the optimal dose for the treatment of recurrent OC based on safety data from prior phase I dose-escalation studies.^13–15^ In a large, multicenter, retrospective cohort study, this combination was shown to be safe with promising oncological response in patients with OC completing three PIPAC cycles.^11^ A decrease in both laparoscopic tumor burden assessed using the peritoneal carcinomatosis index (PCI), and histologic evaluation of tumor biopsies utilizing the peritoneal regression grading score (PRGS) were seen. Additionally, two phase II clinical trials, PIPAC-OV1 and PARROT, demonstrated promising findings in the recurrent setting for OC.^7,8^ PIPAC-OV1 demonstrated an objective response with three partial responses and 30 stable disease cases among 53 patients with recurrent OC; progression-free survival (PFS) was 4.7 months. PARROT demonstrated a median time from ovarian cancer relapse to progression of 12 months among 43 patients with platinum-resistant recurrent OC; however, two intestinal perforations were noted. Currently, only one randomized phase III trial, CTRI2018/08/021223, is ongoing in India comparing three cycles of PIPAC (cisplatin 15 mg/m^2^ and doxorubicin 3 mg/m^2^) versus 6 cycles of standard of care intravenous chemotherapy in platinum-resistant recurrent OC. Preliminary data note an objective response rate of 66.6% in the PIPAC group versus 22.5% in the single-agent intravenous chemotherapy group based on imaging response scored by Response Evaluation Criteria in Solid Tumors (RECIST) version 1.1.^12^

In this study, we evaluated monotherapy PIPAC-CD in patients with recurrent OC in the USA with a phase I trial (US-PIPAC-CD). No systemic therapies were allowed during the trial.

## Materials and Methods

### Ethics statement

This study was conducted according to the principles of the Belmont Report and the Declaration of Helsinki. All patients completed written informed consent, including data and images for publication. This study was approved by the COH institutional review board (IRB 19184), Northwell Health (IRB 20-0859), and the Mayo Clinic (IRB 20-010121).

### Patients

Adult patients aged ≥18 years with histologically confirmed OC with peritoneal carcinomatosis based on cross-sectional imaging or diagnostic laparoscopy who had progressed on at least one evidence-based chemotherapeutic regimen were included if their Eastern Cooperative Oncology Group (ECOG) performance status was ≤2, there were no contraindications to laparoscopic surgery or aerosol therapy, intraoperative laparoscopic findings showed PIPAC access was feasible, there was no evidence of impending bowel obstruction and ≤5 L of ascites, and the patient was not a candidate for cytoreduction and HIPEC. Exclusion criteria included receiving prior maximum cumulative doses of anthracyclines and/or anthracenediones. Of note, patients with gastric and endometrial cancer were also enrolled in this study but were not the subject of the main analysis (results reported in the supplemental material). See Supplemental Table 1 for complete eligibility and exclusion criteria.

### Study Design

This was a nonrandomized, uncontrolled, single-arm, phase I clinical trial without dose escalation to establish the safety of PIPAC-CD in the USA. The rules for accrual were slot limited to not exceed the risk of the traditional 3+3 phase I trial design with modifications to adapt to the patient queue to reduce the time to complete the study.^16,17^ If the PIPAC was not well-tolerated, the plan was to amend the study protocol.

### PIPAC Procedure

During each cycle of PIPAC, a laparoscopic open-entry technique was performed, and two additional laparoscopic ports were placed under direct visualization. Ascites was aspirated and measured. Very limited adhesiolysis was allowed for safe port placement and visual assessment, but no surgical debulking or other interventions were allowed. Visual assessment of tumor burden was recorded using the peritoneal carcinomatosis index (PCI), and biopsies from all accessible quadrants were collected for the peritoneal regression grading score (PRGS). Biopsy sites were selected by the surgeon based on the largest and most suspicious-looking tumor lesions. After biopsies were collected, PIPAC was performed using standardized left lower quadrant port placement, if feasible, with cisplatin 10.5 mg/m^2^ in 150 mL NaCl 0.9% and doxorubicin 2.1 mg/m^2^ in 50 mL NaCl 0.9% delivered using a high-pressure injection (Medrad Stellant injector, Bayer Corporation) and CapnoPen or Nebulizer (Capnomed Corporation, Tubingen, Germany or REGER Medizintechnik GmbH, Villingendorf, Germany, respectively) at an average of 200 psi at 0.5 mL/sec with a maximum of 300 psi. This was followed by 30-min pneumoperitoneum at 12 mmHg containing the aerosolized chemotherapy at room temperature, then release of the pneumoperitoneum and evacuation of the aerosolized chemotherapy. Laparoscopic balloon occlusion ports were used to limit staff exposure to aerosolized chemotherapy. PIPAC was repeated at 4- to 6-week intervals for a planned total of three treatments unless severe adverse events (AEs), dose-limiting toxicity (DLT), disease progression, or patient withdrawal from the study occurred. Patient surveys were used to collect QOL measurements. Patients who experienced clinical benefit from PIPAC were offered up to an additional 3 cycles of PIPAC (total of 6 cycles) on compassionate care exemptions. PIPAC-CD pharmacokinetic information was collected via post-PIPAC serial blood collections and immediate post-PIPAC peritoneal biopsies of normal and tumor tissues.

### Endpoints

The primary endpoint was safety of PIPAC-CD via assessment of DLTs and incidence of treatment-related AEs. AEs were assessed every 4–6 weeks for up to 18 weeks using the Common Terminology Criteria for Adverse Events version 5.0. Following treatment completion (defined as two or more PIPACs, as per convention reported in a large systematic review of PIPAC),^18^ patients were evaluated every 12 weeks. Secondary endpoints evaluated the efficacy of PIPAC utilizing changes in computed tomography (CT) imaging RECIST version 1.1, pathologic PRGS of intraoperative biopsies taken each cycle, and intraoperative PCI. RECIST by CT imaging was evaluated after cycle 2 and at completion of treatment. PCI was calculated by the same surgeon for each patient across all PIPAC cycles received. PRGS is a four-tiered histologic grading system of PM response to chemotherapy, ranging from grade 1 (G1; complete response, with an absence of tumor cells) to grade 4 (G4; no response, with tumor cells showing no regressive features).^19^ PRGS was reported as the average of all quadrant biopsies obtained during each cycle of PIPAC. CA-125 tumor markers were evaluated in patients with OC at the beginning of each cycle. DLTs were defined as delay in >21 days, any G3 or higher nonhematologic toxicity, excluding predefined AEs, that returned to G2 or lower in a specified time period. AEs included G3 nausea, vomiting, abdominal pain, or diarrhea adequately treated and returned to G2 or lower within 48 h, G3 fatigue that returned to G2 or lower within 7 days, G3 metabolic or laboratory abnormalities that were not considered clinically significant and were easily correctable to G2 or lower within 72 h, G3 infusion-related reaction (first occurrence and in the absence of steroid prophylaxis) that resolved within 6 h with appropriate treatment, and G3 neuropathy. DLTs also included Clavien–Dindo surgical complications grade IIIB or higher, G4 thrombocytopenia or neutropenia lasting >7 days, and neutropenic fever or infection. Patient-reported outcomes (PROs) as described by health state and QOL and symptoms were collected before starting treatment, cycles 1–3, and off treatment as measured by the EQ-5D-5L questionnaire, Health-Today, and MD Anderson Symptom Inventory.

### Statistical Analysis

Patient characteristics and clinical results were summarized using descriptive statistics, and survival analysis was calculated using the Kaplan–Meier method. Pharmacokinetic results were reported using mathematical ratios, medians, 95% confidence intervals (CIs), area under the curve (AUC), and standard deviation (SD) (censor date January 2025). The last patient on this arm was enrolled in September 2024.

## Results

### Study schema and patient characteristics

**Fig. 1:**
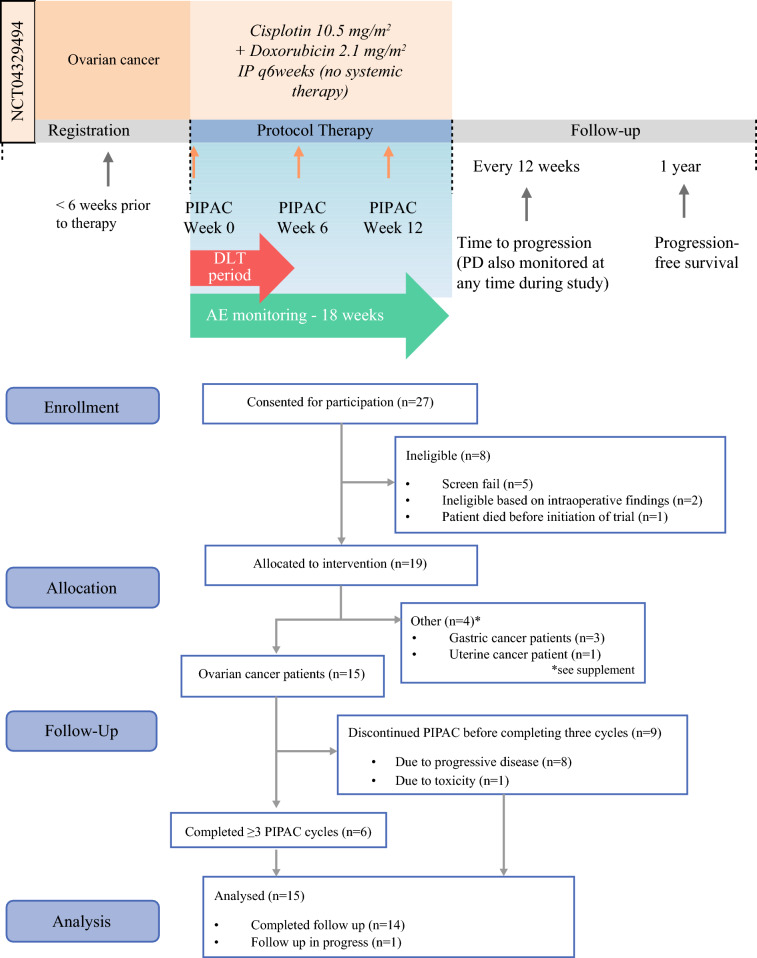
Trial outline: **A** study schema, **B** Consolidated Standards of Reporting Trials CONSORT) flow diagram. AE, adverse event; DLT, dose-limiting toxicity; IP, intraperitoneal; PD, progression of disease; PIPAC, pressurized intraperitoneal aerosolized chemotherapy

Patients with OC received PIPAC cisplatin 10.5 mg/m^2^ and doxorubicin 2.1 mg/m^2^ intraperitoneally every 6 weeks (Fig. 1A). A total of 15 women with OC were enrolled in arm 1 (between September 2020 and September 2024) (Fig. 1B), and the median age was 60.9 years (range 38.3–83.2) (Table 1). Of these, 14 (93.3%) patients had good performance status, with ECOG scores of 0–1, and one patient had an ECOG score of 2. The median prior lines of therapy was 3 (range 1–10). No patients had germline or somatic BRCA mutations.

**Table 1:** Patient demographics and characteristics

Demographic or characteristic	Patients (n=15)
Age at start of treatment	60.9 (38.3–83.2)
Sex	
Female	15 (100.0)
Race	
Asian	2 (13.3)
Caucasian	12 (80.0)
Not disclosed	1 (6.7)
Ethnicity	
Hispanic or Latino	1 (6.7)
Non-Hispanic or Non-Latino	14 (93.3)
Histology at diagnosis	
Low-grade serous carcinoma	5 (33.3)
Clear cell carcinoma	1 (6.7)
High-grade serous carcinoma	6 (40.0)
Hepatoid adenocarcinoma	1 (6.7)
Adenocarcinoma, NOS	2 (13.3)
BRCA mutation	
Germline	0 (0.0)
Somatic	0 (0.0)
ECOG	
0	4 (26.7)
1	10 (66.7)
2	1 (6.7)
Number of prior therapies	3 (1–10)
Baseline disease status	
IP only	4 (26.7)
Extraperitoneal and IP	9 (60.0)
Unknown	2 (13.3)
Stage at enrollment (based on radiographic restaging)	
Stage III	6 (40.0)
Stage IV	7 (46.7)
Unknown	2 (13.3)

Data are presented as median (range) or n (%) unless otherwise indicated.

ECOG, Eastern Cooperative Oncology Group; HIPEC, hyperthermic intraperitoneal chemotherapy; IP, intraperitoneal; NOS, not otherwise specified.

The median baseline PCI was 18. The median baseline PRGS was 2.25. At baseline, nine (69.2%) of the 13 patients with available data had extraperitoneal disease. All patients had previously undergone primary or interval cytoreductive surgery. All except two patients had baseline ascites.

### Feasibility of PIPAC

There were no technical failures during completion of the laparoscopy or PIPAC administration. In total, 13 (86.7%) patients completed at least two cycles of PIPAC (Table 2), including one patient who completed six cycles. Median follow-up was 6.4 months (95% CI 5.4–not reached [NR]). One (6.7%) patient withdrew from the trial after the first PIPAC cycle because of a decline in her baseline ECOG performance status 2 and deterioration of preexisting partial obstructive bowel symptoms. This patient’s withdrawal from the trial was deemed as toxicity related, in the setting of non-compliance to follow-up and lack of imaging to determine disease progression as alternate reason for withdrawal. Eight (53.3%) patients discontinued treatment because of disease progression after the second PIPAC cycle.

**Table 2:** Clinical results

Clinical results	Patients (n=15)
Number of cycles of PIPAC	
1	2 (13.3)
2	7 (46.7)
≥3	6 (40.0)
Patients who received 2 or more cycles	13 (86.7)
Radiographic best response (RECIST)	
PR	1 (6.7)
SD	4 (26.7)
PD	10 (66.7)
Laparoscopic best response (PCI) in patients receiving 2 or more cycles	
Decrease	4 (30.8)
Stable	2 (15.4)
Increase	7 (53.8)
Histologic best response (PRGS) in patients receiving 2 or more cycles	
Decrease	6 (46.2)
Stable	2 (15.4)
Increase	5 (38.5
Off-treatment reason	
Treatment completed per protocol	6 (40.0)
Progression	8 (53.3)
Toxicity	1 (6.7)
Recurrence location	
IP only	3 (20.0)
Extraperitoneal and IP	8 (53.3)
Unknown	3 (20.0)
None	1 (6.7)
Median follow-up months for PFS	6.4 (5.4–NR)
Median PFS months	2.3 (1.7–3.2)
Median OS months	17.1 (5.6–NR)

Data are presented as n (%) or median (95% confidence interval) unless otherwise indicated.

CI, confidence interval; IP, intraperitoneal; NR, not reached; OS, overall survival; PCI, Peritoneal Carcinomatosis Index; PD, progressive disease; PFS, progression free survival; PIPAC, pressurized intraperitoneal aerosolized chemotherapy; PR, partial response; PRGS, Peritoneal Regression Grading Score; RECIST, Response Evaluation Criteria in Solid Tumors; SD, stable disease

### Safety of PIPAC

There were no Clavien–Dindo surgical complications. There were no DLTs. There were 16 G2 or higher toxicities (two G3, 14 G2) that were attributable to the treatment (possible/probably/definite) (Table 3). The G3 toxicities were abdominal pain and anorexia. The three most common toxicities were abdominal pain, fatigue, and nausea. Of note, there were no G4/5 AEs.

**Table 3:** Adverse events grade ≥2

Adverse event	Grade 2	Grade 3
Abdominal pain	3 (20.0)	1 (6.7)
Fatigue	2 (13.3)	
Flank pain	1 (6.7)	
Hypomagnesemia	1 (6.7)	
Hypotension	1 (6.7)	
Lymphocytopenia	1 (6.7)	
Nausea	2 (13.3)	
Vomiting	1 (6.7)	

Data are presented as n (%)

### Efficacy of PIPAC

**Fig. 2:**
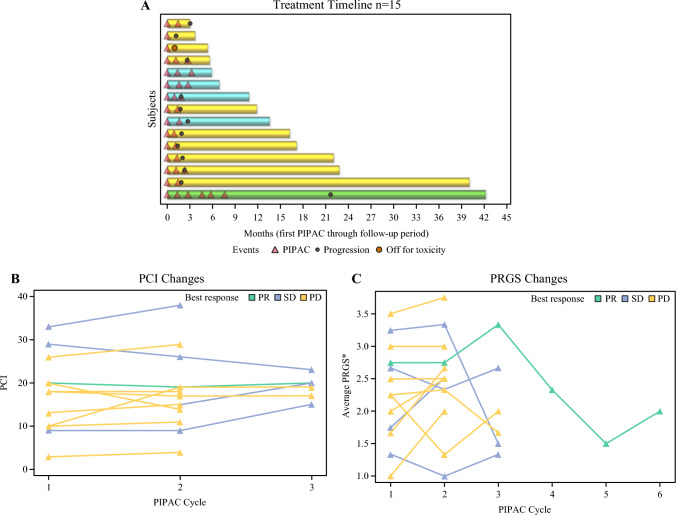
Response to pressurized intraperitoneal aerosolized chemotherapy (PIPAC) treatments with best response measured by computed tomography imaging using Response Evaluation Criteria in Solid Tumors (RECIST). **A** Swimmer plot of each patient and best response to treatment. Green bars indicate patients with partial response (PR), blue bars indicate stable disease (SD), and yellow bars indicate progressive disease. **B** Laparoscopic peritoneal carcinomatosis index (PCI) changes between cycles for each patient. Green lines indicate patients with PR (n=1), blue lines indicate SD (n=4), and yellow lines indicate progressive disease (n=8). **C** Histologic response by changes in average Peritoneal Regression Grading Score (PRGS) over each PIPAC cycle. Green lines indicate patients with PR (n=1), blue lines indicate SD (n=4), and yellow lines indicate progressive disease (n=8). PD, progression of disease

**Fig. 3.**
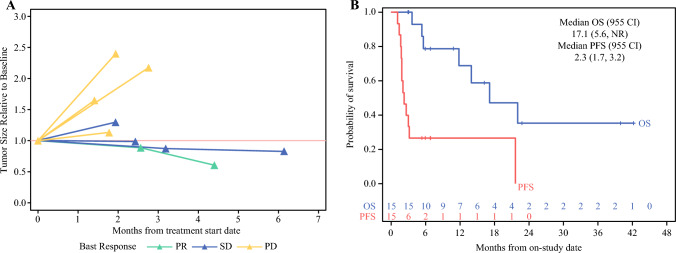
**A** Tumor growth patterns by computed tomography (CT) imaging of target lesions (n=8). Available target lesion data from seven patients included four (one stable disease [SD]) with non-target or new lesions only, and three with baseline measurements only. **B** Survival of patients with ovarian carcinoma (n=15) after pressurized intraperitoneal aerosolized chemotherapy with cisplatin 10.5 mg/m^2^ and doxorubicin 2.1 mg/m^2^. Median progression-free survival (PFS) (red) was 2.3 months (95% confidence interval [CI] 1.7–3.2) and median overall survival (OS) (blue) was 17.1 months (95% CI 5.6–not reached [NR]). PD, progression of disease; PR, partial response

Response to PIPAC was evaluated by CT imaging using RECIST, laparoscopic PCI, and histologic PRGS. Based on the best overall response using RECIST criteria, four patients had stable disease (26.7%), and one patient (6.7%) had a durable partial response that persisted for 14.0 months after completing 6 PIPAC cycles (Figs. 2A and 3A Table 2). Among the 13 patients in the OC cohort who completed at least two of the planned cycles of PIPAC, six (46.2%) had a stable PCI or a decrease (Fig. 2B). Each patient had the same surgeon across all of the PIPAC cycles they received. The amount of ascites between cycles 1 and 2 was stable or decreased in 12 (92.3%). Mean PRGS between cycles 1 and 2 was stable or had a histologic response in eight (61.5%) (Fig. 2C). Median PFS was 2.3 months (95% CI 1.7–3.2), and median OS was 17.1 months (95% CI 5.6–NR) (Fig. 3B).

### Pharmacokinetics

**Fig. 4:**
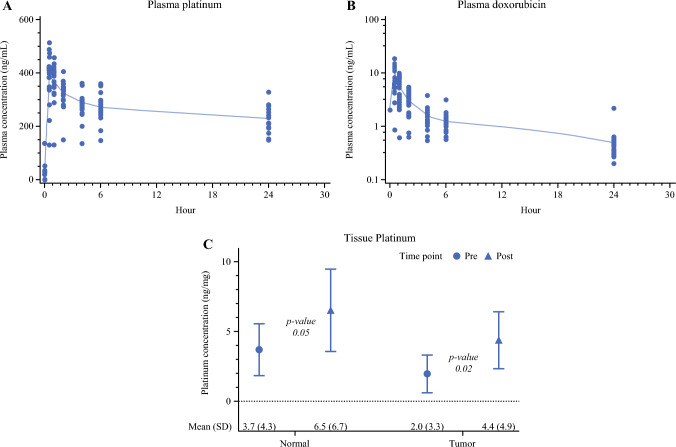
Pharmacokinetic data of serum and tissue chemotherapy: **A** Cisplatin plasma concentration (n=9 patients) over 24 h; **B** doxorubicin plasma concentration (n=12 patients) over 24 h; **C** platinum tissue concentration in normal and tumor pre- and post-pressurized intraperitoneal aerosolized chemotherapy specimens. All patients/cycles with both pre- and post-treatment values by tissue type were included. A 1-sided T-test was used to test the overall difference between pre- and post-treatment within each tissue type

Cisplatin pharmacokinetic data were available for nine patients, and doxorubicin pharmacokinetic data were available for 12 patients. Across all PIPAC procedures, the average cisplatin maximum plasma concentration (C_max_) was 404.6 ± SD 78.8 ng/mL, with an average AUC in the first 24 h after PIPAC administration of 6418.0 ± SD 1198.3 ng × h/mL (Fig. 4A). The average doxorubicin C_max_ was 9.9 ± SD 4.4 ng/mL with an AUC in the first 24 h after PIPAC administration of 33.6 ± SD 12.6 ng × h/mL (Fig. 4B). The mean tissue platinum concentration in normal and tumor biopsies increased significantly after PIPAC (p = 0.05 and p = 0.02, respectively).  Post-PIPAC normal and tumor tissue biopsies were taken immediately after PIPAC administration, and measured 6.5 ± SD 6.7 ng/mg and 4.4 ± SD 4.9 ng/mg, respectively (Fig. 4C).

### Patient-Reported Outcomes

Seven patients completed the three different questionnaires. Six of the seven patients had multiple timepoints reported. Among these six patients, the values remained stable, and there were no statistical differences between different timepoints (Supplemental Fig. S1).

## Discussion

Our multi-institutional trial demonstrated the feasibility of PIPAC across three US academic centers. PIPAC-CD was safe and well tolerated in patients with OC, with a G3 AE rate of 13.3% and no Clavien–Dindo complications, consistent with prior PIPAC-CD studies.^7–12^ The PIPAC completion rate (defined as at least 2 cycles per convention among PIPAC trials) was 86.7%.^18^

Limited efficacy signals were derived in this trial of PIPAC monotherapy in patients with recurrent OC. Overall PFS was 2.3 months. In patients completing at least 2 PIPAC cycles, intraperitoneal overall response by a decrease in PCI was 30.8%, and this was stable in 15.4% of patients. There was one partial response and four stable disease responses by RECIST.

In our study, 76.9% of patients with OC had extraperitoneal disease at baseline, including lung and liver metastases, in contrast to previously published OC PIPAC trials. With a high proportion of extraperitoneal metastases, a high rate of progression in the extraperitoneal areas was observed in our trial. Intraperitoneal tumor control was similar to that in previous trials, given similar intraperitoneal response rates seen by laparoscopic and histologic measures (PCI, PRGS). Additionally, our trial included a more heavily pretreated OC population than other OC PIPAC trials, which may explain the differences in disease control rate (33.3% in our trial vs 62% in PIPAC OV-1, 82% in PARROT, and 85% in the phase III Indian trial).^7,9,12^ Generally, cross-trial comparisons are hampered by differences in patient characteristics, patient eligibility, baseline characteristics, and timing of evaluation of disease status, which result in different survival expectations and outcomes. Therefore, differences in PFS could also be attributed to the timing of RECIST evaluation or definition of clinical benefit. The PIPAC OV-1 trial included a similarly heavily pretreated population to that in our trial, with a median of 3 (range 2–8) prior lines and reported OS of 10.9 months and PFS of 4.7 months. In comparison, our trial reported OS of 17.1 months and PFS of 2.3 months. The RECIST evaluation took place after cycle #2 in our trial, whereas RECIST was evaluated after cycle #3 in PIPAC OV-1. The PARROT trial enrolled a less heavily pretreated OC population with median prior lines of 1 (range 1–2) with a time to progression of 12 months, but no laparoscopic responses except for stable disease (89%), and a high rate of no histologic response (70%) were noted.^8^ In contrast, our trial demonstrated a 30.8% response by laparoscopic PCI, and 15.4% stable disease, though differences may be due to different laparoscopic scores (Sugarbaker PCI vs Fagotti score), and different patient populations (prior lines of therapies, extraperitoneal disease). The reported 1.0 month (range 0–11) median time to progression from the last PIPAC application in the PARROT trial is similar to our findings, as the majority of our patients experienced progression shortly after their last PIPAC cycle. One exception in our trial was an exceptional responder: a patient with low-grade serous OC who experienced a 14-month time interval without disease progression.^17^ Although OS of 27 months was longer in the PARROT trial than the 17.1 months OS reported in our trial, subsequent therapies following PIPAC may confound this measure, especially if there were inherent differences in the timing of PIPAC during the natural progression of OC, and thus inherently different a priori survival outcomes.

Evaluation of the response to treatment is not uniform across all PIPAC trials. RECIST, laparoscopic scores such as PCI or the Fagotti Score, and PRGS histologic grading scoring are variably used for endpoints in PIPAC trials, with inherent benefits and limitations to each modality. The presence and extent of PM is not easily visible or scored via RECIST by CT, or magnetic resonance imaging. RECIST can be affected by variability in tumor size measurements both intra- and inter-observer as well as differences in tumor growth and patterns present within each patient.^20^ In contrast, laparoscopic scoring (PCI and Fagotti) allows for direct visualization of the tumors, including small implants or miliary disease not visible on imaging. However, the ability to evaluate the entire peritoneal cavity is often limited by adhesive disease, which may lead to an underestimation of the true nature of metastatic spread,^21^ as well as subjective scoring by the surgeon. Histologic grading systems such as PRGS allows for microscopic evaluation for assessment of treatment response, which may not be evident using imaging or laparoscopic evaluation due to the presence of inflammation or fibrosis affecting tumor size measurements.^19^ Unfortunately, the major limitation of histologic evaluation is the bias of intraoperative surgeon choice of biopsy location. Moreover, both laparoscopic and histologic scoring evaluate disease only in the intraperitoneal cavity, without assessing disease in the retroperitoneum (lymphatic metastases) or extraperitoneal areas (e.g., parenchymal liver metastases, pleural or mediastinal disease), which afflict the majority of patients with recurrent OC.

The histologic intraperitoneal treatment response to PIPAC in our trial noted a 61.5% stable or decrease in PRGS, versus a 29.6% stable PRGS response; with no patients showing a decrease in PRGS in the PARROT trial. In contrast, 85% of patients with recurrent OC in the Indian trial demonstrated a PRGS response, though this trial accrued patients with less heavily pretreated OC with a median of one prior line.^8,12^ The PIPAC OV-1 trial used a slightly different histologic grading system but reported 62% moderate to strong tumor regression.^7^ Laparoscopic scoring (via PCI or Fagotti) in patients who received ≥3 cycles was decreased or stable in 50% of patients in our trial, compared to 88.9% in PARROT, and 76% in PIPAC OV-1. No other study looked at change in ascites volume following treatment, but our study saw 92.3% of patients with stable or decreased ascites.

PROs from PIPAC were limited because of the poor overall completion of PRO questionnaires by patients, which is a common barrier in assessing PROs in palliative trials, and no statistical differences were seen throughout the study.

Our study confirmed previous pharmacokinetic findings for cisplatin and doxorubicin in PIPAC.^22,23^ We additionally measured tissue platinum concentrations through immediate pre- and post-PIPAC sampling and demonstrated significant increases in both normal and tumor samples.

This study was limited by a small sample size of 15 patients with OC. None of the patients with OC in this trial carried germline or somatic BRCA 1 or 2 mutations. Although the best responder was a patient with low-grade serous OC with a durable partial response, three other patients with low-grade serous OC either progressed or had stable disease on trial. Histologic subtype does not appear to be a predictive characteristic. The optimal patient selection and timing of PIPAC treatment in the recurrent setting will need to be determined through further study. Nonetheless, given the generally safe and well-tolerated nature of PIPAC, combined with the promising results seen in our study, along with PARROT, PIPAC OV-1, and the phase III Indian trial, PIPAC deserves further study to optimize its treatment potential for recurrent OC.

Most PIPAC monotherapy trials to date have excluded patients with extraperitoneal disease as there is limited treatment effect in these disease sites with intraperitoneal delivery of low-dose chemotherapy. This trial further demonstrated the lack of extraperitoneal disease response with PIPAC monotherapy. Most patients with baseline extraperitoneal disease demonstrated extraperitoneal progression of disease. Systemic concentration levels of cisplatin and doxorubicin were significantly lower than for PIPAC compared with historical systemic levels for intravenous chemotherapy, normothermic intraperitoneal chemotherapy, or HIPEC.^24–26^ Given these findings, combining intravenous chemotherapy or other systemic therapies with PIPAC for multimodal therapy may address the shortcomings of PIPAC monotherapy and should be explored. Additionally, although the combination of cisplatin and doxorubicin has been the most commonly used PIPAC drug regimen in OC, they do not represent the most active drugs in platinum-resistant recurrent OC. Other chemotherapy drugs have been evaluated in PIPAC studies and may represent more active therapies in platinum-resistant OC. Of these drugs, nab-paclitaxel is a promising candidate for multimodal PIPAC therapy in recurrent OC, because of the significant activity demonstrated by its intravenous administration in platinum-resistant disease, as well as a slow clearance from the intraperitoneal cavity.^27,28^ The combination of intravenous and PIPAC nab-paclitaxel in recurrent OC is currently being studied in the USA and may represent a more active therapeutic combination in patients with OC with peritoneal and extraperitoneal metastases.^29^

In conclusion, PIPAC-CD was feasible, safe, and well tolerated in patients with heavily pretreated OC who were not candidates for cytoreductive surgery. PIPAC monotherapy with cisplatin and doxorubicin appears to demonstrate a significant intraperitoneal response with reduced peritoneal metastatic burden by laparoscopic and histologic scoring. However, the majority of patients with recurrent OC have extraperitoneal metastases, which drive their survival outcomes, and therefore limit the ultimate efficacy of PIPAC monotherapy. Although intraperitoneal disease control is important in recurrent OC, and PIPAC represents a step forward in managing this disease, the future lies in combining this novel therapy with multimodal intravenous chemotherapy to control extraperitoneal tumor burden and in identifying the optimal chemotherapies best suited for platinum-resistant OC.

## Supplementary Information

Below is the link to the electronic supplementary material.

Supplementary file1 (DOCX 625 KB)
