# Supplementary material for: Safety and Efficacy of Cisplatin and Doxorubicin Pressurized Intraperitoneal Aerosolized Chemotherapy (PIPAC) in Patients with Ovarian Cancer with Peritoneal Metastases: A Multicenter US Phase I Trial
Source: Ann Surg Oncol. 2025 Sep 30;33(1):415–25. doi: 10.1245/s10434-025-18432-0 (PMC12689763; doi:10.1245/s10434-025-18432-0)
Supplement: Supplementary file 1 — Supplementary file1 (DOCX 625 KB) [file 10434_2025_18432_MOESM1_ESM.docx]

**Supplemental Material**

**Supplemental Table 1. Eligibility Criteria**

| Inclusion Criteria:   - Documented informed consent of the participant and/or legally authorized representative - Patients must have histologically confirmed ovarian, uterine, gastric, appendiceal or colorectal cancer with peritoneal carcinomatosis - Prior IP chemotherapy is permitted - ECOG performance status ≤ 2 - Absolute neutrophil count (ANC) ≥ 1500/mm^3^ - Platelets ≥ 100,000/mm^3^ - Hemoglobin ≥ 9g/dL - Serum total bilirubin ≤ 1.5x upper limit of normal (ULN) - Alanine aminotransferase (ALT), serum glutamate pyruvate transaminase (SGPT), and aspartate aminotransferase (AST), serum glutamic oxaloacetic transaminase (SGOT) ≤ 2.5x ULN, unless liver metastases are present or unless patient is known to have chronic liver disease (hepatitis) in which case AST and ALT must be ≤ 5x ULN - Alkaline phosphatase ≤ 2x ULN - Serum creatinine ≤ 1.5x ULN, or creatinine clearance ≥ 40 mL/min as calculated by the Cockcroft-Gault formula - No contraindications for a laparoscopy - The peritoneal disease does not have to be measurable by RECIST 1.1 but needs to be visible on cross sectional imaging or diagnostic laparoscopy - Patients must have progressed on at least one evidence-based chemotherapeutic regimen - For patients with a known history of chronic hepatitis B virus (HBV) infection, the HBV viral load must be undetectable on suppressive therapy, if indicated - Patients with a known history of hepatitis C virus (HCV) infection must have been treated and cured. For patients with HCV infection who are currently on treatment, they are eligible if they have an undetectable HCV viral load - Women of childbearing potential (WOCBP) and male patients with WOCBP partner must be using an adequate method of contraception to avoid pregnancy throughout the study and for up to 12 weeks after the last dose of investigational product in such a manner that the risk of pregnancy is minimized. WOCBP include any female who has experienced menarche and who has not undergone successful surgical sterilization (hysterectomy, bilateral tubal ligation, or bilateral oophorectomy) or is not postmenopausal. Post menopause is defined as:   - Amenorrhea ≥ 12 consecutive months without another cause or   - For women with irregular menstrual periods and on hormone replacement therapy (HRT), a documented serum follicle stimulating hormone (FSH) level > 35 mIU/mL   - Women who are using oral contraceptives, other hormonal contraceptives (vaginal products, skin patches, or implanted or injectable products), or mechanical products such as an intrauterine device or barrier methods (diaphragm, condoms, spermicides) to prevent pregnancy, or are practicing abstinence or where their partner is sterile (e.g., vasectomy) should be considered to be of childbearing potential - INCLUSION TO PROCEED WITH PIPAC: Laparoscopy findings must meet all of the below criteria in order to proceed to PIPAC:   - PIPAC access is feasible   - There is room for aerosol therapy   - There is no evidence of impending bowel obstruction   - ≤ 5L of ascites   - Not a candidate for cytoreduction and HIPEC |
| --- |
| Exclusion Criteria:   - Previous treatment with maximum cumulative doses of doxorubicin, daunorubicin, epirubicin, idarubicin, and/or other anthracyclines and anthracenediones |

**Supplemental Table 2. Demographics and Characteristics of Gastric and Endometrial Cancer Patients**

| Demographic or Characteristic | Non-OC patients (n=4) |
| --- | --- |
| Median age at start of treatment (range) | 58.2 (45.4 – 69.1) |
| Gender |  |
| Female | 4 (100%) |
| Race |  |
| Asian | 1 (25%) |
| Caucasian | 2 (50%) |
| Not disclosed | 1 (25%) |
| Ethnicity |  |
| Hispanic or Latino | 1 (25%) |
| Non-Hispanic or Non-Latino | 3 (75%) |
| Disease site |  |
| Gastric | 3 (75%) |
| Uterine | 1 (25%) |
| Histology at diagnosis |  |
| Adenocarcinoma, NOS | 1 (25%) |
| Adenocarcinoma, metastatic, NOS | 1 (25%) |
| Malignant neoplasm, NOS | 1 (25%) |
| Signet ring cell carcinoma | 1 (25%) |
| ECOG |  |
| 0 | 1 (25%) |
| 1 | 3 (75%) |
| 2 | 0 (0%) |
| Number of prior therapies, [median (range)] | 2 (2 – 2) |
| Baseline disease status |  |
| IP only | 4 (21.1%) |
| Extraperitoneal and IP | 11 (57.9%) |
| Unknown | 4 (21.1%) |

OC, ovarian cancer; Non-OC patients include gastric cancer (n=3) and endometrial cancer (n=1); NOS, not otherwise specified; ECOG, Eastern Cooperative Oncology Group; IP, intraperitoneal; HIPEC, hyperthermic intraperitoneal chemotherapy

**Supplemental Table 3. Clinical Results of Gastric and Endometrial Cancer Patients**

| Clinical Results | Non-OC patients (n=4) |
| --- | --- |
| Number of Cycles of PIPAC |  |
| 1 | 1 (25%) |
| 2 | 3 (75%) |
| 3 (or more) | 0 (0%) |
| Patients who received 2 or more cycles | 3 (75%) |
| Radiographic best response (RECIST) |  |
| PR | 0 (%) |
| SD | 1 (25%) |
| PD | 3 (75%) |
| Laparoscopic best response (PCI) in patients receiving 2 or more cycles (n=3) |  |
| Decrease | 0 (0%) |
| Stable | 0 (0%) |
| Increase | 3 (100%) |
| Histologic best response (PRGS) in patients receiving 2 or more cycles (n=3) |  |
| Decrease | 1 (33%) |
| Stable | 1 (33%) |
| Increase | 1 (33%) |
| Off-Treatment Reason |  |
| Treatment completed per protocol | 0 (0%) |
| Progression | 3 (75%) |
| Toxicity | 1 (25%) |
| Recurrence Location |  |
| IP only | 1 (25%) |
| Extraperitoneal and IP | 1 (25%) |
| Unknown | 2 (50%) |
| None | 0 (0%) |
| Median follow-up months for PFS (95%CI)* | NR (NR, NR) |
| Median PFS months (95%CI) | 1.7 (1.1, NR) |
| Median OS months (95%CI) | 8.4 (NR, NR) |

*All patients progressed (Kaplan-Meier approach not needed as there is no censoring). Non-OC patients include gastric cancer (n=3) and endometrial cancer (n=1); PIPAC, pressurized intraperitoneal aerosolized chemotherapy; RECIST, Response Evaluation Criteria in Solid Tumors; PR, partial response; SD, stable disease; PD, progressive disease; PCI, Peritoneal Carcinomatosis Index; PRGS, Peritoneal Regression Grading Score; IP, intraperitoneal; PFS, progression free survival; OS, overall survival; NR, not reached

**Supplemental Table 4. Adverse Events Grade ≥ 2 in Gastric (n=3) and Endometrial Cancer Patients (n=1)**

| **Adverse Event** | **Grade 2** | **Grade 3** |
| --- | --- | --- |
| Anorexia |  | 1 (25%) |
| Fatigue | 1 (25%) |  |
| Nausea | 1 (25%) |  |


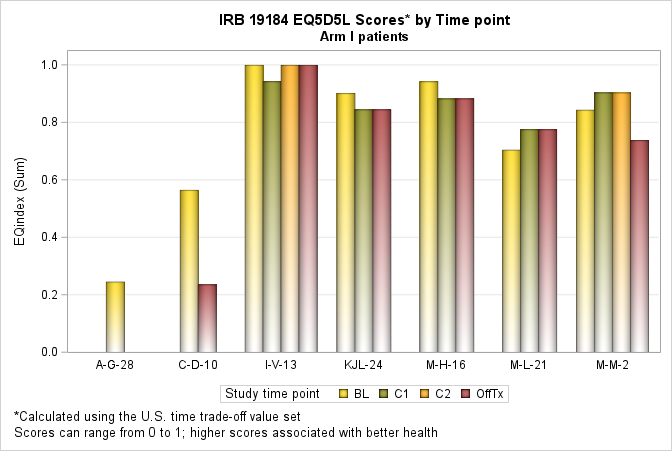

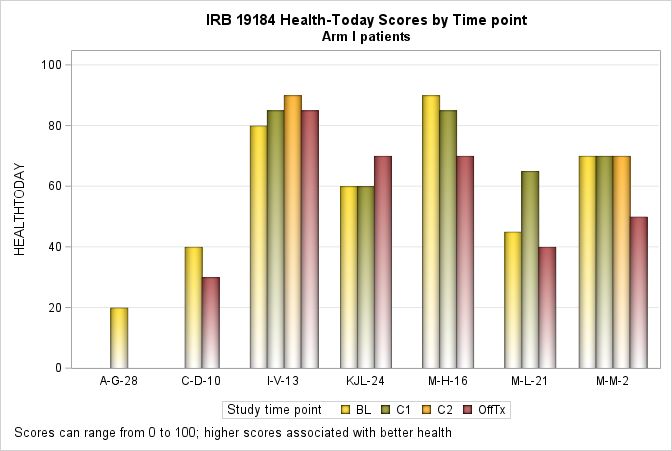

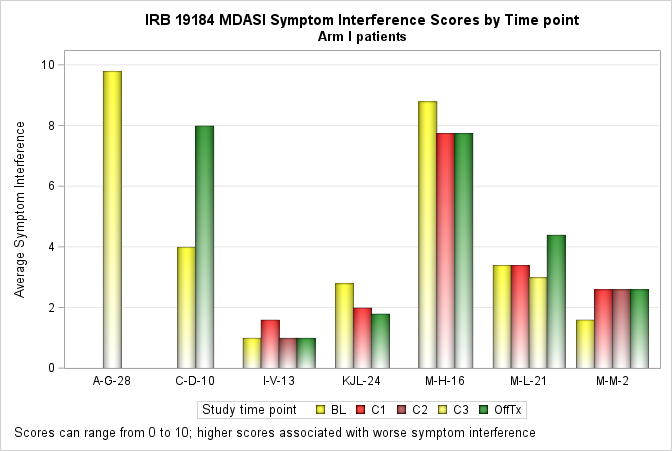

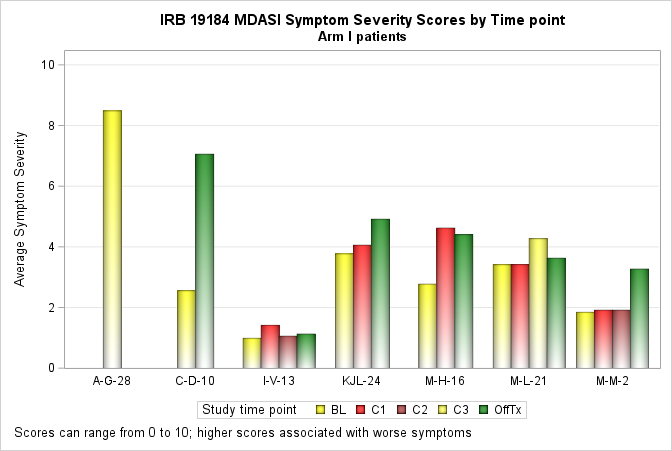


A

B

C

D

**Supplemental Figure 1. Patient-Reported Outcomes.** Patient reported outcomes as measured by A) EQ5D5L; B) Health-Today; C) MDASI Symptom Severity; and D) MDASI Symptom Interference scores. EQ5D5L, European Quality of Life Five Dimension Five Level Scale; MDASI, MD Anderson Symptom Inventory
